# Estrogen receptor α enhances the transcriptional activity of ETS-1 and promotes the proliferation, migration and invasion of neuroblastoma cell in a ligand dependent manner

**DOI:** 10.1186/s12885-015-1495-3

**Published:** 2015-06-30

**Authors:** Peng Cao, Fan Feng, Guofu Dong, Chunyong Yu, Sizhe Feng, Erlin Song, Guobing Shi, Yong Liang, Guobiao Liang

**Affiliations:** 1 Department of Neurosurgery, Institute of Neurology, General Hospital of Shenyang Military Area Command, Shenyang Northern Hospital, 83 Wenhua Road, Shenhe District, Shenyang City, Liaoning Province 110016 PR China; 2 Department of Pharmacy, General Hospital of Shenyang Military Area Command, Shenyang Northern Hospital, 83 Wenhua Road, Shenhe District, Shenyang City, Liaoning Province 110016 PR China; 3 Institute of Radiation Medicine, Military Medical Science Academy of the Chinese PLA, 27 Taiping Road, Beijing City, 100850 PR China; 4 Department of Urology, General Hospital of the Chinese PLA, 28 Fuxing Road, Beijing City, 100853 PR China; 5 Key Laboratory of Cardiovascular Medicine Research, Ministry of Education, Harbin Medical University, Harbin, 150081 PR China

## Abstract

**Background:**

It is well known that estrogen receptor α (ERα) participates in the pathogenic progress of breast cancer, hepatocellular carcinoma and head and neck squamous cell carcinoma. In neuroblastoma cells and related cancer clinical specimens, moreover, the ectopic expression of ERα has been identified. However, the detailed function of ERα in the proliferation of neuroblastoma cell is yet unclear.

**Methods:**

The transcriptional activity of ETS-1 (E26 transformation specific sequence 1) was measured by luciferase analysis. Western blot assays and Real-time RT-PCR were used to examine the expression of ERα, ETS-1 and its targeted genes. The protein-protein interaction between ERα and ETS-1 was determined by co-IP and GST-Pull down assays. The accumulation of ETS-1 in nuclear was detected by western blot assays, and the recruitment of ETS-1 to its targeted gene’s promoter was tested by ChIP assays. Moreover, SH-SY5Y cells’ proliferation, anchor-independent growth, migration and invasion were quantified using the MTT, soft agar or Trans-well assay, respectively.

**Results:**

The transcriptional activity of ETS-1 was significantly increased following estrogen treatment, and this effect was related to ligand-mediated activation of ERα. The interaction between the ERα and ETS-1 was identified, and enhancement of ERα activation would up-regulate the ETS-1 transcription factor activity via modulating its cytoplasm/nucleus translocation and the recruitment of ETS-1 to its target gene’s promoter. Furthermore, treatment of estrogen increased proliferation, migration and invasion of neuroblastoma cells, whereas the antagonist of ERα reduced those effects.

**Conclusions:**

In this study, we provided evidences that activation of ERα promoted neuroblastoma cells proliferation and up-regulated the transcriptional activity of ETS-1. By investigating the role of ERα in the ETS-1 activity regulation, we demonstrated that ERα may be a novel ETS-1 co-activator and thus a potential therapeutic target in human neuroblastoma treatment.

## Background

Estrogen is one of the key regulators of the development and progression of several cancers, such as breast cancer [1–6]. In mammalian cells, estrogen is recognized by estrogen receptors (ERs) [1]. Among these nuclear receptors, ERα contains a ligand-independent activation function domain 1 (AF-1 domain) in N-terminal and an AF-2 domain in C-terminal, and a DNA binding domain (DBD domain) in between [2]. In cell nucleus, ERα modulates the expression of estrogen response genes via binding to ERE (estrogen responsive element) sequence on their promoter [1–3]. The cross-talk between ERα and EGFR (Epidermal growth factor receptor) pathway has been reported in lung cancer, esophagus cancer and neck squamous cell carcinoma [4]. Recently, expression of ERα has been identified in neuroblastoma cells [5]. Several studies showed that ERα crosstalks with IGF-IR in regulating proliferation of neuroprotection and neuroblastoma [6]. However, the detailed function of ERα in the proliferation, migration or invasion of neuroblastoma cells has not been uncovered.

The transcription factor ETS-1 (E26 transformation specific sequence 1) belongs to ETS protein family [7]. It contains an ETS domain (transcription activation domain) and a helix DNA-binding domain [7]. ETS family is involved in the regulation of cancer cells’ proliferation, development, apoptosis, metastasis, invasion and angiogenesis [7]. High level of ETS-1 was identified in breast cancer, ovarian cancer and cervical carcinoma [8]. In nucleus, ETS-1 regulates expression of several target genes, such as MMP1, MMP9, u-PA and c-Met, via binding to ETS-binding site (EBS, the 5′-GGAA/T-3′ sequence motif) within the promoter regions of those genes in presence of hepatocyte growth factor (HGF) [8]. Some co-regulators participate in ETS-1 activity, such as SRC-1 (steroid receptor coactivator 1), AIB-1 (amplified in breast cancer1) and NCoR [8, 9]. Myers et al., 2009 and Kalet et al., 2013 provided the evidences that ETS-1 would modulate the activity of ERα and promoted the proliferation of breast cancer via ERα response genes [8, 9]. It is valuable to declare the interaction between ETS-1 and ERα.

Several evidences also demonstrated that transcription factors or nuclear receptors could crosstalk in a feedback way [10–12]. For example, aryl hydrocarbon receptor (AHR) can up-regulate ER signaling through protein-interaction [10]; whereas ER can also repress AHR target genes’ transcription [11]. Given that ERα could enhance the expression of MMPs [12], we therefore decided to examine whether ERα could modulate ETS-1’s activity in neuroblastoma, an ERα positive human cancer. In this study, we found that ERα interacts with ETS-1 in neuroblastoma cell. Transcriptional activity of ETS-1 was significantly increased when ERα had been activated by estrogen. Estrogen mediated ERα activation significantly promoted the proliferation, migration and invasion of neuroblastoma Cell. Our results suggested that ERα would enhance ETS-1’s activity via promoting its cytoplasm/nucleus translocation, recruiting ETS-1 to the EBS of ETS-1 responsible gene’s promoter in a ligand dependent manner.

## Methods

### Plasmids

The sequences of ETS-1 or ERα with or without FLAG sequence was generated by PCR amplification from vectors contain full length sequences (Origene Company, USA) and cloned into pcDNA3.1 plasmids. Luciferase reporter genes, *mmp1*, *mmp9*, *c-Met* and *uPA* [13], EBS (GGAT) 8 sequences were synthesized by using chemical synthesis methods (Gene Ray Company, Shanghai, China) and were cloned into pGL4.26 plasmid. The expression vectors of SRC-1 and AIB-1 were also obtained from Origene Company, USA. The siRNA targeted to ERα or ETS-1 was obtained from Santa Cruz Biotech Company, USA. The expression vectors of NCoR and SMRT were gift from Dr. Jiajun Cui [14]. All vectors were confirmed by DNA sequencing.

### Cell culture and reagents

ARQ-197 (c-Met inhibitor) was descripted in reference [15]. E2 (the agonist of ERα, 17-β-estradiol) and ICI-182780 (the antagonist of ERα) were from Sigma (St. Louis, MO, USA), and other agents (Amersham Biosciences, Piscataway, NJ, USA) were used. Agents were configured to 10 mM DMSO solution, stored in 4 °C. Recombinant human HGF was obtained from Pepro-Tech (Rocky Hill, NJ, USA). Human neuroblastoma cell line SH-SY5Y (ERα positive) and breast cancer cell line MDA-MB-231 (ERα negative), were from cell resources center of Chinese Academy of Medical Sciences & Peking Union Medical College in China. Cells were cultured in complete Dulbecco’s modified Eagle’s medium (DMEM) (Invitrogen, Carlsbad, CA) in a sterile incubator maintained at 37 °C with 5 % CO_2_. HEK293 cells were obtained from American Type Culture Collection (ATCC), and were cultured in Roswell Park Memorial Institute 1640 (RPMI1640) medium (Invitrogen, Carlsbad, CA) in a sterile incubator maintained at 37 °C with 5 % CO_2_.

### Stable transfection

SH-SY5Y cells were transfected with empty vector, ETS-1 vector, ERα vector, control siRNA, ETS-1 siRNA or ERα siRNA; and MDA-MB-231 cells were transfected with empty vector or ERα vector by using Lipofectamine 2000 (Invitrogen, Carlsbad, CA). Then, transfected cells were cultured in 200–500 μg/ml G418 (Invitrogen, Carlsbad, CA) for approximately 2 months. Individual clones were screened by Western Blotting analysis using anti-ETS1 or anti-ERα antibody. Similar results were observed with stable transfection or transient transfection, the individual clones or pool clones.

### Luciferase assay

SH-SY5Y and MDA-MB-231 cells were seeded in 24-well plates (Corning, NY, USA) in phenol red-free DMEM (Gibco, Grand Island, NY, USA) supplemented with 0.5 % charcoal-stripped FBS (Hyclone, Logan, UT, USA). Transfection was performed using Lipofectamine 2000 (Invitrogen, Carlsbad, CA). Cells were co-transfected with luciferase reporters and then harvested for analysis of luciferase and β-galactosidase activities following protocols descripted in reference [16]. The luciferase assays were performed without or with indicated concentration of E2, ICI-182780, ARQ-197 or HGF. Similar results were obtained from three independent experiments.

### RNA isolation and real-time RT-PCR

Total RNA was extracted using the PARISTM Kit (Applied Biosystems, Foster City, CA) according to the manufacturer’s instructions. Multiscribe TM Reverse Transcriptase (Applied Biosystems, Foster City, CA) was used to synthesize the complementary DNA templates. Real-time reverse transcription–polymerase chain reactions were performed in an Applied Biosystems 7500 Detection system using Maxima SYBR Green/ROX qPCR Master Mix Assays (Fermentas, USA) following reference [17, 18]. The housekeeping gene β-Actin was chosen as the loading control. The expression of targeted genes’ mRNA was determined from the threshold cycle (Ct), and relative expression levels were normalized to the expression of human β-Actin mRNA and calculated by the 22^-△△^ Ct method. Primers which used in real-time RT-PCR were listed in Table 1.

**Table 1:** Real-time RT-PCR Primers

Target genes	Primers
MMP1	Forward primer: 5′-aagccatcacttaccttgcact-3′
	Reverse primer: 5′-tcagagaccttggtgaatgtca-3′
MMP9	Forward primer: 5′-ctggagacctgagaaccaa-3′
	Reverse primer: 5′-actgctcaaagcctccacaaga-3′
β-Actin	Forward primer: 5′-ctccatcctggcctcgctgt-3′
	Reverse primer: 5′-gctgtcaccttcaccgttcc-3′

**Table 2:** The dose-effect of agents on ETS-1′s transcriptional activity

Agents	*IC*_*50*_/*EC*_*50*_ (nM)	*IC*_*max*_/*EC*_*max*_ (μM)	*R*^*2*^ Value	*P* Value
E2	18.75 ± 1.22	0.10	0.94	0.0024
HGF	6.22 ± 0.75 (ng/ml)	0.03	0.95	0.0098
ICI-182780	26.53 ± 4.15	0.10	0.92	0.015
ARQ-197	17.75 ± 3.66	0.30	0.91	0.0044

### Antibodies and immunoblotting analysis (western blotting)

Antibodies against ERα, ETS-1, MMP1, MMP9, SRC-1, AIB-1, Lamin A/C, β-Actin and GAPDH were obtained from Santa Cruz Biotechnology (Santa Cruz Biotech, CA, USA). Antibodies against NCoR and SMRT were gift from Dr. Jiajun Cui and descripted in reference [14]. A polyclonal anti-rabbit IgG antibody and anti-Flag monoclonal antibody both conjugated with the horseradish peroxidase (HRP) were from Sigma (St. Louis, MO, USA). SH-SY5Y or MDA-MB-231 cells were seeded and cultured in six-well plates (Corning, NY, USA). The cells, which were treated with indicated concentration compounds or transfected with vectors, were harvested by RIPA buffer supplemented with protease inhibitors cocktails (Sigma, Louis, MO). Total protein samples were performed by SDS-PAGE and trans-printed to poly-vinylidene fluoride (PVDF) membranes (Millipore, Billerica, MA). Then, membranes were blocked with 10 % BSA in TBST buffer and then incubated 2 h at 37°Cwith rabbit primary antibody against human ERα (1:1,000); rabbit primary antibody against ETS-1 (1:2000); mouse primary antibody against human MMP1 (1:500), MMP9 (1:1000), SRC-1 (1:1000), AIB-1 (1:1000); rabbit primary antibody against human NCoR (1:500) or SMRT (1:500) and mouse primary monoclonal antibody against human GAPDH diluted in TBST containing 10 % BSA and subsequently washed three times in TBST for 5 min each. Then membranes were incubated with the HRP-conjugated secondary antibodies (1:5000) after washed three times in TBST for 5 min each. At last, the blot was developed with enhanced chemiluminescence reagents (Pierce, USA) by X-ray films. When incubating HRP-Flag monoclonal antibody (1:5000), the blots were visualized without incubating secondary antibody. The blots were performed on three independent occasions with similar results.

### Immunoprecipitation

SH-SY5Y cells were transfected with FLAG-ERα or FLAG-ETS-1 using Lipofectamine 2000. Then, cells were harvested and lysed in the immunoprecipitation buffer after 18–24 h culture at 4 °C. The Co-IP analyze was performed with anti-FLAG monoclonal antibody (Sigma-Aldrich, USA) and then detected by immunoblotting assays treated without or with 100nM E2 following the protocols descripted in reference [19, 20].

### GST-pull down assay

ERα or ETS-1 was expressed as GST-fusion proteins in Escherichia coli (*E. coli*) strain DH5α and bound to the glutathione-Sepharose beads purified as described by the manufacturer (Amersham Biosciences). The expression plasmid for FLAG-ERα or FLAG-ETS1 was used for the expression in HEK293 cells and purified by FLAG-beads. FLAG-ERα or FLAG-ETS-1 was incubated with GST alone, GST-ETS-1 or GST-ERα fusion protein bound to glutathione-Sepharose beads in 500 μl of binding buffer at 4 °C for 4 h. The beads were precipitated, washed three times with binding buffer, and subjected to SDS-PAGE and WB (western blot) assays.

### ChIP

The recruitment of transcriptional factor (ETS-1) or nuclear receptor (ERα) to its DNA binding elements was analyzed by ChIP assays as protocols described previously [15, 19, 21]. SH-SY5Y cells were transfected with plasmids or treated with indicated compounds, and fixed by adding formaldehyde to the medium. After cross-linking, glycine was added at a final concentration of 125 mM, and the cells were harvested with lysis buffer. The cell nuclei sub-fractions were pelleted by centrifugation and resuspended in nuclear lysis buffer. The nuclear lysates were sonicated to generate DNA fragments of 0.5-1 kb, and then ChIP assays were performed with antibodies against ERα, ETS-1, SRC-1, AIB-1, NCoR or SMRT. Real-time PCR amplification was performed with DNA extracted from the ChIP assay and primers flanking the ETS binding elements in promoter region of mmp1 gene.

The primers used in ChIP analysis were as follows [13]: *mmp1* gene’s promoter forward:’-TTCCAGCCTTTTCATCATCC-3′; reverse: 5′-CGGCACCTGT ACTGACTGAA-3′; Input Genomic DNA forward: 5′-AACCTATTAACTCA CCCTTGT-3′ Input Genomic DNA reverse: 5′-CCTCCATTCAAAAGATCTTATTATTTAGCATCTCCT-3′

### Subcellular fractionation

The localization of ERα and ETS-1 was determined by the subcellular fractionation assays following the protocol descripted in reference [22]. Briefly, SH-SY5Y cells were homogenized using a Dounce homogenizer and the homogenate was centrifuged at 366 g for 10 min. Next, the pellets were analyzed as the nuclear fraction. The supernatant was centrifuged again at 13201 g for 10 min, and the final supernatant was analyzed as the cytoplasmic fraction. Then, IB analysis was performed. Anti-β-Actin rabbit antibody (1:5000) was used to detect the cytoplasmic fraction, and anti-Lamin A/C mouse antibody (1:2500) was used to detect the nucleus fraction.

### Cell proliferation assays

Cell proliferation was analyzed by MTT-assay as described previously [23]. The proliferation of SH-SY5Y cells was determined using a Cell Titer 96® nonradioactive cell proliferation assay kit (Promega, USA), according to the manufacturer’s instructions. Cells, which were transfected with plasmids or treated with agents, were seeded into 96-well plate and incubated at 37 °C with 5 % CO2. After incubating for 1 day, 2 days, 3 days, 4 days and 5 days, cells were harvested and analyzed. Finally, growth curves for each cell group were drawn according to the volume of O.D. 490 nm from the 96-well plate reader. The MTT cell growth assays were performed for three independent times.

### Anchorage-independent growth assay

SH-SY5Y cells were treated with agents. Cells were plated on six-well plates (500 per well) (Corning, Corning, NY), with a bottom layer of 0.7 % low-melting-temperature agar in DMEM and a top layer of 0.25 % agar in DMEM. Colony number was the mean ± SD of three independent experiments scored after 3–4 weeks of growth [23].

### Trans-well invasion and migration assay

The invasion and migration assays were performed in 24-well plates using the trans-well chamber (Corning, NY, USA) fitted with a polyethylene terephthalate filter membrane with 8-μm pores. For invasion assay, the membrane undersurface was coated with 30 μl ECM (Extracellular matrix) gel from Engelbreth-Holm-Swarm mouse sarcoma (BD Biosciences, Bedford, MA, USA) mixed with RPMI-1640 serum free medium in 1:5 dilution for 4 h at 37 °C. The top chambers of the trans-wells were filled with 0.2 ml of cells (5 × 105 cells/ml) in serum-free medium, and the bottom chambers were filled with 0.25 ml of RPMI 1640 medium containing 10 % FBS. The cells were incubated in the trans-wells at 37 °C in 5 % CO2 for 4 h or 24 h. The relative invading cells were measured following the methods descripted in reference [4]. Values were corrected for protein concentration and are presented as the mean ± SD of three independent experiments, each with two samples per experimental treatment [24]. The mean values were obtained from three replicate experiments.

### Ethics statement

Our studies are in compliance with the Helsinki Declaration. Our work aims to declare the cross-talk between transcriptional factors and the underlying molecular mechanisms. We did not use any materials from clinical specimens. And the methods did not relate to the clinical trial or methods. Only the cell lines used in this work were obtained from the typical biological sample preservation Center but not clinical specimens, human subjects, human material or data.

### Statistical analysis

The WB results were analyzed by the ALPHA INNOTECH analysis software. The relative expression level was calculated: (indicated group protein expression level / loading control expression level) / (control group protein expression level / loading control expression level). All statistical significance analyses were performed using SPSS statistical software. *P-*value of <0.05 was considered statistical significant. Statistical significance in the luciferase activity and cell growth assays was analyzed by Bonferroni correction with or without two ways ANOVA. The R2, P and EC50/IC50 values were calculated by Origin 8.5 software.

## Results

### Estrogen enhances the transcriptional activity of ETS-1

**Fig. 1:**
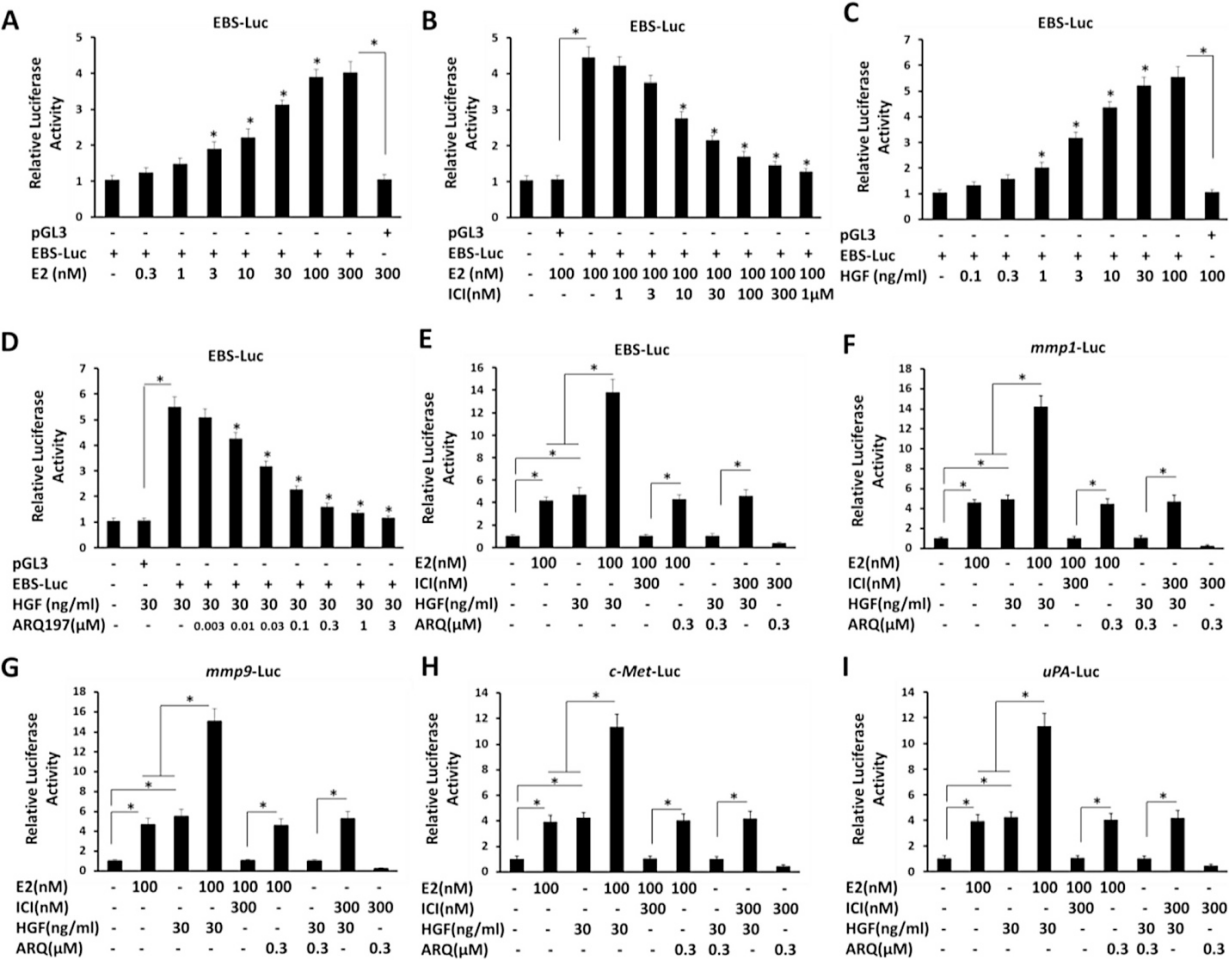
The effect of estrogen on ETS-1 transcriptional activity. SH-SY5Y cells were co-transfected with EBS (**a**-**e**), *mmp1* (**f**), *mmp9* (**g**), *c-Met* (**h**) and *uPA* (**i**) reporters; then treated with indicated concentration of E2 (17-β-estradiol, the agonist of ERα), ICI-182780 (the antagonist of ERα), HGF (hepatocyte growth factor, the agonist of c-Met) or ARQ-197 (the antagonist of c-Met). Cells were harvested and determined by the Luciferase assays. The values are the mean ± SD from three independent experiments. * P < 0.05

To discover the role estrogen plays in regulating the transcriptional activity of ETS-1, a common endogenous estrogen E2 was employed in luciferase assays. SH-SY5Y cells were co-transfected with ETS-1 binding site EBS-Luc reporters. E2 increased the activity of ETS-1 in a dose-dependent manner (Fig. 1a, Table 2), the *EC*_*50*_ value is 18.75 ± 1.22nM. The antagonist of ERα ICI-182780 down-regulated ETS-1’s activity induced by E2 (Fig. 1b, Table 2), the *IC*_*50*_ value is 26.53 ± 4.15nM. To confirm the activity of ETS-1 in SH-SY5Y cells, the agonist (HGF) and antagonist (ARQ-197) of ETS-1 signaling pathway were used. As shown in Fig. 1c and d, HGF increased the EBS-Luc reporter activity in a dose dependent manner, the *EC*_*50*_ value is 6.22 ± 0.75 ng/ml; whereas ARQ-197 inhibited the EBS-Luc activity induced by HGF, the *IC*_*50*_ value is 17.75 ± 3.66nM. These all indicated that ERα increased the activity of ETS-1 in a ligand dependent manner.

Next, the potential cross-talk of ERα and ETS-1 was detected. SH-SY5Y cells were co-transfected with EBS-Luc, or ETS-1 responsive genes *mmp1*, *mmp9*, *c-Met* and *uPA* luciferase reporters and harvested and analyzed by luciferase assays. As shown in Fig. 1e-i, both E2 and HGF synergistically enhanced the activity of EBS-Luc, MMP1-Luc and MMP9-Luc. ICI-182780 inhibited the effect of E2 but not HGF; whereas ARQ-197 almost blocked HGF’s effect but not E2. Moreover, ICI-182780 did not reduce the effect of HGF on ETS-1 activity. Suggest both estrogen and HGF regulate ETS-1 activity independently.

**Fig. 2:**
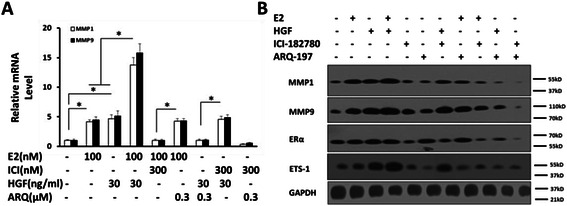
The effect of estrogen and HGF on the expression of ETS-1 targeted genes. SH-SY5Y cells were treated with indicated concentration of E2, ICI-182780, HGF or ARQ-197. **a** Identification of ETS-1 responsive genes’ mRNA level by Real-time RT-PCR assays. Cells were treated with indicated concentration of agents, and then be examined by RT-PCR assays. **b** The protein level of ETS-1, MMP1/9 and ERα was identified by Western blot. The values are the mean ± SD from three independent experiments. * P < 0.05

Then, the transcription and expression level of MMP1/9 was tested by RT-PCR and western blot. As shown in Fig. 2a and b, E2 and HGF synergistically enhanced the mRNA level and protein level of MMP1 and MMP9. ICI-182780 blocked the effect of E2, but not HGF; whereas ARQ-197 inhibited the effect of HGF but not E2. Moreover, ICI-182780 did not reduce the activity of HGF and the antagonist of these two pathways synergistically reduced the expression of those ETS-1 response genes. These results indicated that ERα activation may up-regulate the expression of ETS-1 targeted genes independent of HGF/c-Met signaling, and the enhancement of ETS-1 activity induced by E2 would be mediated by ERα independently.

### The specificity of estrogen mediated ETS-1 activity regulation

**Fig. 3:**
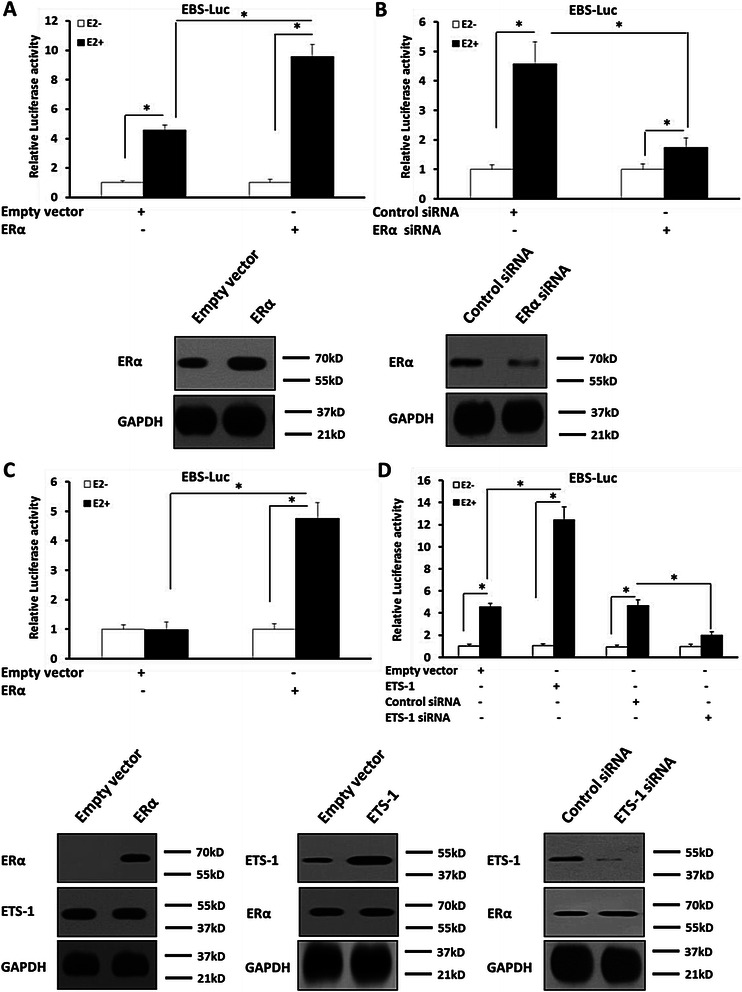
ERα but not the HGF/c-Met mediated the enhancement of ETS-1 activity induced by estrogen. **a**,**b** Cells were treated with 100nM E2 (the ECmax concentration of estrogen). The SH-SY5Y cells were stably transfected with empty vector (**a**), ERα vectors (**a**), control siRNA (**b**,**d**), ERα siRNA (**b**), ETS-1 vector (**d**) or ETS-1 siRNA (**d**); whereas MDA-MB-231 cells were stably transfected with empty vector (**c**) or ERα vectors (**c**). Then, cells which were co-transfected with EBS-Luc reporters and harvested for the Luciferase analysis. The expression of ERα and ETS-1 were determined by immunoblots, and the results were showed at the panels at the bottom of the figure. The values are the mean ± SD from three independent experiments. * P < 0.05

To study the specificity of estrogen on regulating ETS-1 activity, SH-SY5Y cells, which expresses ERα (Fig. 3a and b), were stably transfected with empty vector, ERα, control siRNA, or ERα siRNA for ERα overexpression and knock-down. Overexpression of ERα enhanced the activity of EBS-Luc reporter activity only in the presence of E2 (Fig. 3a). Knock-down of endogenous ERα dramatically decreased the activity of the EBS-Luc reporters, activated by E2, in SH-SY5Y cells compared with control (Fig. 3b). These data indicated that ERα itself is required for the effect of E2 on ETS-1 activity. Human breast cancer cells MDA-MB-231, which lacks the ERα but normally expresses ETS-1, were co-transfected with the EBS-Luc, ERα or empty vector. As shown in Fig. 3c, in presence of E2, stable expression of ERα but not empty vector enhanced the transcriptional activity of ETS-1 for 4.3-folds. This result further showed that ERα regulates the transcriptional activity of ETS-1 induced by estrogen.

Next, the involvement of ETS-1 in ERα-mediated transcription needs to be examined. Overexpression of ETS-1 increased the activity of EBS-Luc (Fig. 3d); whereas this activity activated by E2 decreased dramatically in the down-regulation of endogenous ETS-1′s (Fig. 3d) protein level via its siRNA in SH-SY5Y cells. These results indicated estrogen mediated induction of ERα leads to up-regulation of ETS-1 transcriptional activity, and finally increases expression of ETS-1 downstream genes, such as MMP1/9 in an ETS-1 dependent manner.

### ERα interacts with ETS-1 in an estrogen-dependent manner

**Fig. 4:**
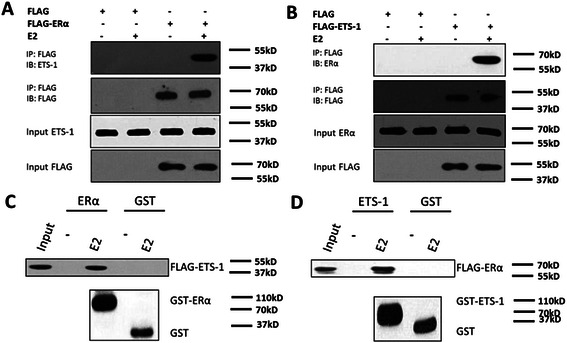
ERα can interact with ETS-1. **a**-**b** Interaction of endogenous ERα, or ETS-1 with exogenous FLAG-ETS-1, or FLAG-ERα. FLAG-tagged ERα (**a**) or FLAG-tagged ETS-1 (**b**) or FLAG empty vector (**a**-**b**) was transfected into SH-SY5Y cells. Cell lysates were immunoprecipitated by anti-FLAG monoclonal antibody, and the precipitates were then immunoblotted with anti-ETS-1 or anti-ERα antibody. **c**-**d***In vitro* interaction of ETS-1 with ERα. Glutathione-Sepharose beads bound with GST-ERα (**c**), GST-ETS-1 (**d**) or with GST (**c**-**d**) were incubated with purified FLAG-labeled ETS-1 or ERα in the presence or absence of 100nM E2. After washing the beads, the bound proteins were eluted and subjected to SDS-PAGE and IB assays

Following our previous observation that ETS-1 interacts with ERα, detailed study was performed. SH-SY5Y cells were transfected with the FLAG-ERα or FLAG empty plasmid. Then the co-immunoprecipitation (co-IP) and immunoblotting (IB) assays were performed. The results showed that FLAG-ERα interacted with the endogenous ETS-1 (Fig. 4a) in the presence of E2. From converse co-IP assay, we showed that FLAG-ETS1 interacted with endogenous ERα (Fig. 4b) in E2-dependent manner. To determine whether ETS-1 interacts with ERα directly, the purified GST-ERα or GST-ETS1 was incubated with purified FLAG-ETS1 or FLAG-ERα for GST pull-down assays. The results showed that GST-ERα interacts with FLAG-ETS1 (Fig. 4c) and GST-ETS1 interacts with FLAG-ERα (Fig. 4d). Taken together, these observations indicated that ETS-1 binds to ERα directly, suggested that E2 may regulate ETS-1′s activity via ERα/ETS-1 interaction.

### Effect of estrogen on ETS-1′s cytoplasm/nuclear translocation

**Fig. 5:**
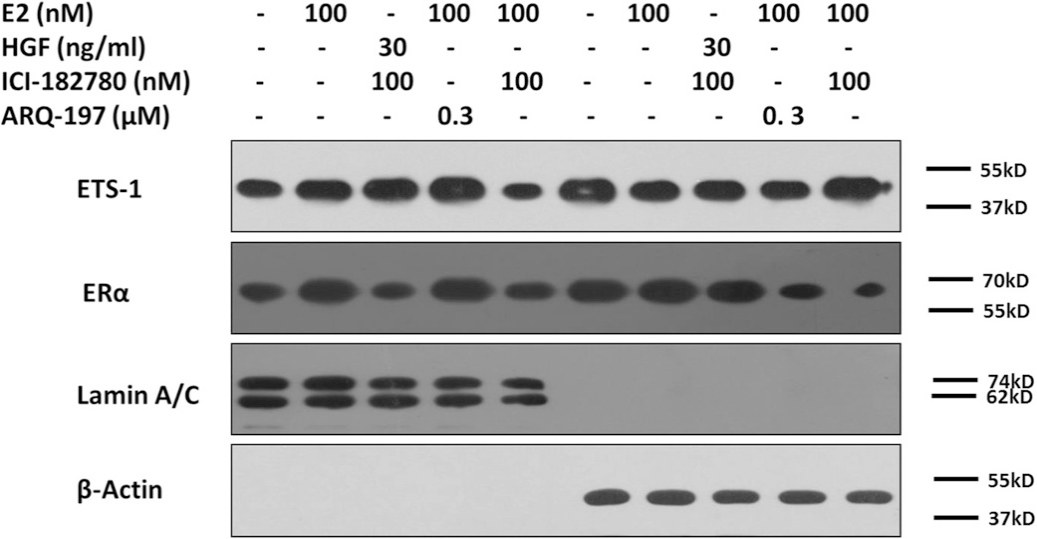
Effect of E2 on ETS-1 cytoplasm/nucleus translocation. SH-SY5Y cells were treated with indicated amount of E2, ICI-182780, or ARQ-197. Then, cells were fractionated into the cytoplasmic fractions and nucleus fractions. The fractions were detected with ETS-1 and ERα antibodies. The Lamin A/C was used as the nucleus indicator. The ß-actin was used as the cytoplasmic marker

Following the protein-interaction results, it is necessary to investigate the detailed mechanism of ERα-mediated ETS-1 activity regulation. SH-SY5Y cells were treated with E2, ICI-182780 or ARQ-197. Then, cells were collected and separated into cytoplasmic/nuclear subcellular fractions, and ERα or ETS-1 was detected by western blot. As shown in Fig. 5, ERα and ETS-1 could be detected in both the cytoplasm and nuclear fractions. E2 increased the proportion of ERα and ETS-1 in the nuclear (Fig. 5). ICI-182780 disrupted the E2 induced cytoplasm/nuclear translocation of ERα and ETS-1 (Fig. 5). ARQ-197 did not modulate the effect of E2 on ETS-1′s translocation (Fig. 5). After treating ICI-182780, a tiny reduction of ERα could be observed than that in breast cancer cells; it might due to the cell type specificity and not be a common phenomenon due to genetic background of SH-SY5Y cells different from breast cancer cells. Those results are in accord with the former findings and suggest ERα would regulate ETS-1 activity via altering its cytoplasm/nuclear translocation dependent to E2 but independent to HGF/c-Met.

### Effect of estrogen on the *mmp1*′s promoter recruitment of ETS-1

**Fig. 6:**
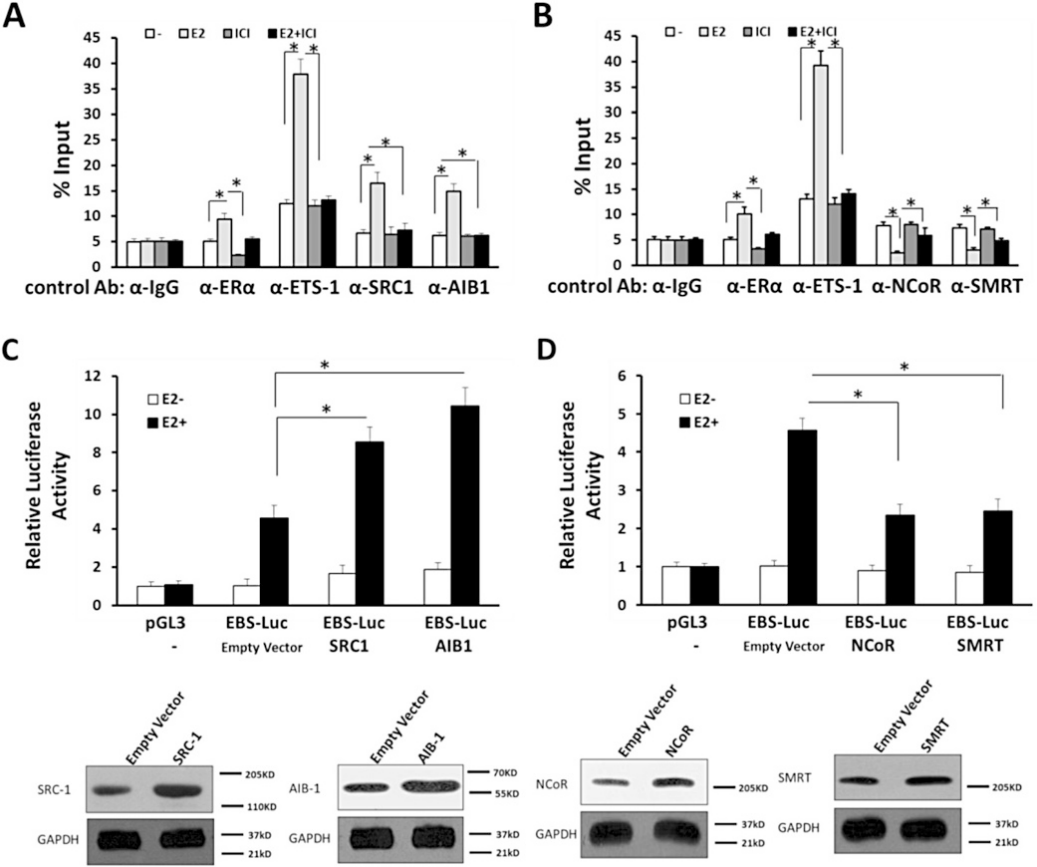
Estradiol modulated the recruitment of ETS-1 and transcriptional co-regulator to *mmp1* promoter region. **a** The recruitment of ETS-1, ERα, SRC-1 and AIB-1 to the mmp1 promoter was detected by ChIP assay. **b** The recruitment of ETS-1, ERα, NCoR and SMRT to the *mmp1* promoter was detected by ChIP assay. (**c**-**d**) SH-SY5Y cells were stimulated with 10nM E2 for 1 h. SH-SY5Y cells were transfected with SRC-1 (**a**), AIB-1 (**a**), NCoR-1 (**b**), or SMRT (**b**) expression vectors or empty vectors. Cells were then harvested for the luciferase assay. The values are the mean ± SD from three independent experiments. Western blot (bottom) indicates the expression level of proteins with anti-SRC1, anti-AIB1, anti-NCoR, or anti-SMRT antibodies. GAPDH was used as loading control. *P < 0.05

To further investigate regulatory activity of estrogen on ETS-1, we performed ChIP assays. Binding of ETS-1 at the *mmp1* promoter, which contains the EBS, was detected by ChIP. As expected, NCoR, SMRT, ETS-1, ERα, SRC-1 and AIB-1 were recruited to the mmp1 promoter (Fig. 6a and b). In addition, E2 potentiated the recruitment of ERα, ETS-1, SRC-1 or AIB-1 to *mmp1* promoter; whereas ICI-182780 down-regulated this effect (Fig. 6a). Meanwhile, E2 also reduced the recruitment of NCoR and SMRT to the promoter (Fig. 6B), which are negative transcriptional regulators of nuclear receptors.

We next studied whether these transcriptional regulators participate in this estrogen-ETS-1 axis. SH-SY5Y cells were co-transfected with SRC-1, AIB-1, NCoR or SMRT plasmids, and then treated without or with E2. As shown in Fig. 6C and D, activity of ETS-1 induced by E2 was enhanced by transfection of SRC-1 or AIB-1 vectors, and reduced after transfection of NCoR or SMRT vectors. These results suggested that estrogen would enhance the recruitment of ETS-1 and transcription factor co-regulators to the downstream gene’s promoter region.

### ERα Increases proliferation of SH-SY5Y Cells

**Fig. 7:**
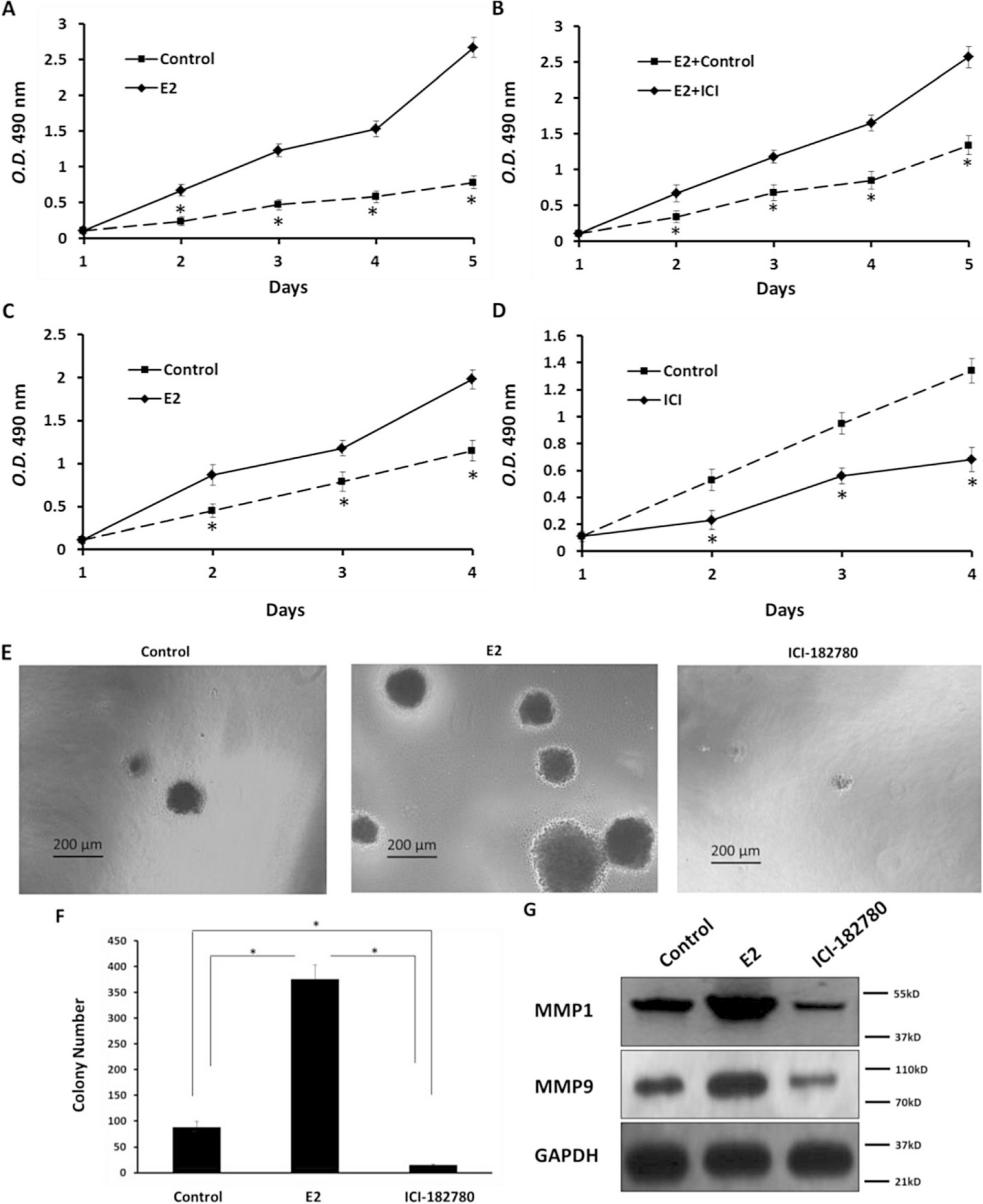
Effect of estrogen and ERα on SH-SY5Y cells proliferation and anchor-independent growth. SH-SY5Y cells, which were cultured in phenol red-free DMEM added 2 % charcoal-stripped FBS (**a** and **b**) or in normal DMEM added 10 % normal FBS (**c** and **d**), were treated with E2 (100nM) or ICI-182780 (300nM). Cells were then measured by MTT assay (**a**-**d**) or soft agar assay (**e**). Colony was shown in the photographs (**e**). (**a**-**d**, **f**) Data are mean ± SD of triplicate independent experiments and have been repeated 3 times with similar numbers. The effect of Estrogen on ETS-1 targeted genes MMP1 or MMP9 was detected by Western blot (**g**). *P < 0.05 versus Solvent control (DMSO) or E2; *P < 0.05 versus Solvent control (DMSO) or ICI-182780; *P < 0.05 versus with E2 or ICI-182780

To study whether ERα activation enhances SH-SY5Y cells proliferation, we performed MTT, trans-well, and soft agar assays. For MTT-assays, SH-SY5Y cells were cultured in phenol red-free DMEM added 2 % charcoal-stripped FBS (Fig. 7a and b) or in normal DMEM added 10 % normal FBS (Fig. 7c and d). As shown in Fig. 7, up-regulation of ERα activity markedly enhanced the proliferation ability of SH-SY5Y cells, while down-regulation of ERα activity induced by E2 markedly reduced SH-SY5Y cells growth. Treatment of E2 promoted the proliferation of SH-SY5Y cells and ICI-182780 down regulated the growth of SH-SY5Y cells.

Next, the role of ERα on SH-SY5Y cell’s anchor-independent growth was examined. ERα’s activation markedly enhanced SH-SY5Y cell growth (Fig. 7e and f). Impairment of ERα activation reduced cell proliferation (Fig. 7e and f). These data showed that estrogen participates in cell anchor-independent growth or invasion.

**Fig. 8:**
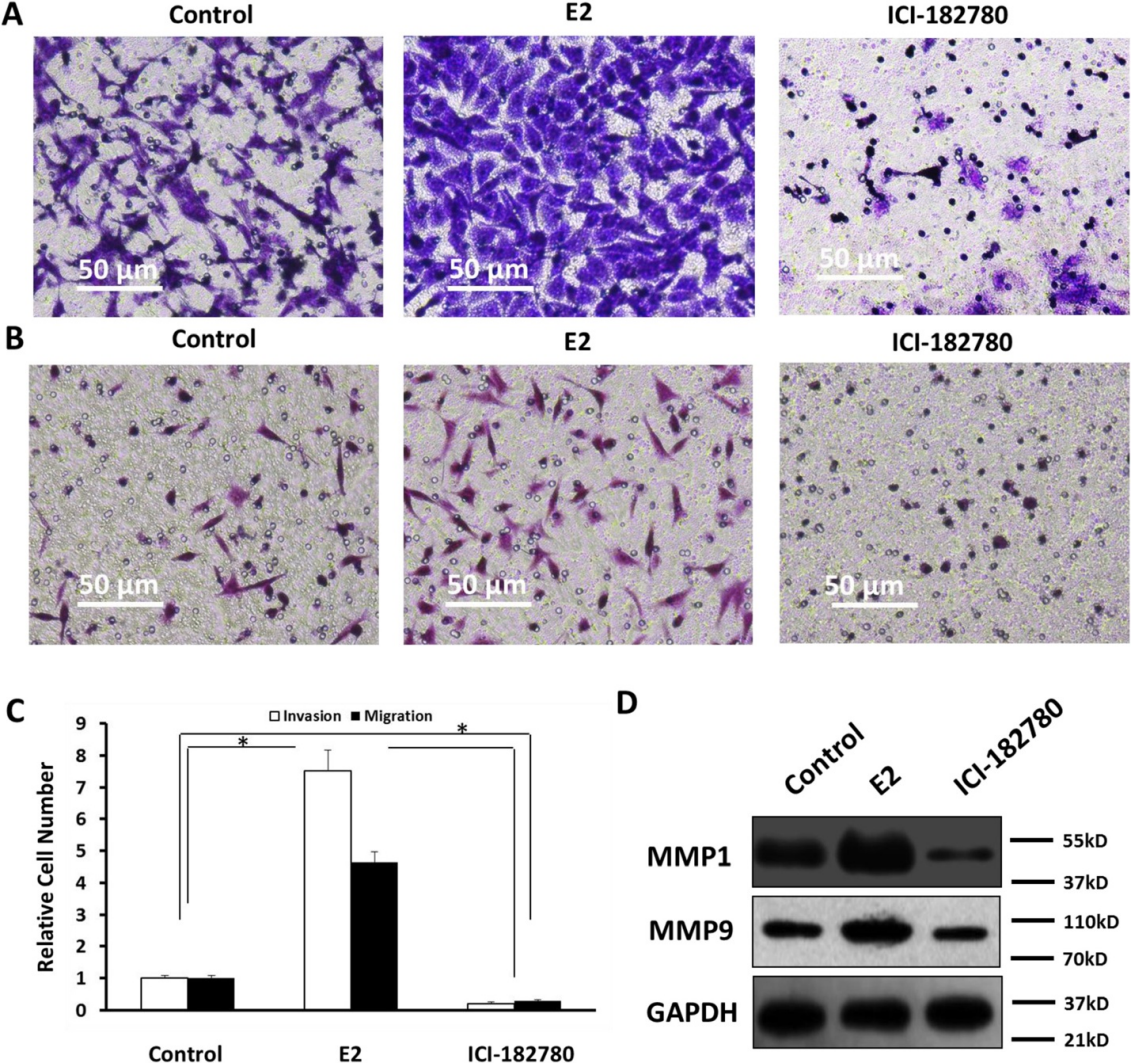
Effect of estrogen and ERα on SH-SY5Y cells invasion and migration. **a**-**d** SH-SY5Y cells were treated with E2 (100nM), or ICI-182780 (300nM). Cells were then measured by trans-well assays. The results were showed in the photographs (**a** and **b**) and (**c**) mean ± SD of triplicate independent experiments and have been repeated 3 times with similar numbers. The effect of Estrogen and ERα on ETS-1 targeted genes MMP1 or MMP9 was detected by WB assay (**d**). *P < 0.05

Moreover, the effect of ERα activity on SH-SY5Y cell’s invasion and migration was examined. Up-regulation of ERα’s activity markedly enhanced SH-SY5Y cell invasion and migration (Fig. 8a,b,d). Our data showed that estrogen increased the expression of ETS-1 targeted genes MMP1/9, which participated in cell migration or invasion (Fig. 8c). Taken together, ERα activation promoted the SH-SY5Y cell’s proliferation, anchor-independent growth, invasion and migration in a ligand-dependent manner.

## Discussion

In this study, we identified the nuclear receptor/transcription factor ERα as an ETS-1 interacting protein and regulator. The protein-interaction between ERα and ETS-1 has been validated by *in vitro* and *in vivo* assays, including co-immunoprecipitation or GST pull-down. ERα activated by its agonist increased the transcriptional activity of ETS-1 and the expression of ETS-1 responsive genes MMP1/9. In contrast, impairment of ERα activation via its antagonist reduced ETS-1′s activity. Moreover, the effect of ERα on ETS-1 was further examined in MDA-MB-231 and SH-SY5Y cells, revealed that ERα mediates the induction of ETS-1 induced by estrogen E2. Moreover, exogenous E2 stimulated neuroblastoma cell proliferation, migration and invasion. We also showed a positive regulatory feedback in E2/ETS-1 signaling that E2 mediated activation of ERα increase ETS-1 activity and ETS-1 protein level. We hypothesis that E2 mediated increasing  of ETS-1 level is one of the downstream effects that ensure the accessibility of the signaling.

ETS-1 is a transcription factor, which has been implicated as a downstream effector of HGF/c-Met signaling pathway [25]. In nucleus, ETS-1 mediates transcription via binding to the ETS binding sequence (EBS) in promoter/enhancer regions of targeting gene [25]. HGF would induce expression of ETS-1 target genes include the ETS-1, MMPs, urokinase-type plasminogen activator, growth factors and the growth factor receptor like c-Met or HER2 [25–27]. Accumulating evidences have shown that ETS-1 could interact with several co-regulators, including co-activators or co-repressors. The transcriptional activity of ETS-1 was modulated by these co-regulators. Sequence-motif LxxLL in Loop 1 of ETS domain has been identified to the recognition site for co-regulators binding, such as SRC/p160 [28, 29]. The p160 family of steroid co-regulator was thought to be exclusively associated with nuclear receptors and some steroid-independent transcription factors, including NK-kB, AP1, P53, ER81, ETS-1 and ETS-2 [20]. Since ERα is a ligand-dependent nuclear receptor, ERα mediated stimulation of cancerous cells proliferation requires estrogen, such as E2 [30–34]. We showed that ERα could efficiently enhance ETS-1 transcriptional in the SH-SY5Y cells were cultured in phenol red-free medium with charcoal dextran-treated fetal bovine serum only supplemented estrogen. Therefore, ERα itself was required for the activity of ETS-1′s transcriptional activity induced by E2. Moreover, ERα would be trans-located into nucleus in respond to estrogen [33] and binds to the genome DNA of the estrogen responsive element (ERE) sequences to regulate the expression of downstream genes [34]. Combine with our observations that estrogen induced the accumulation of ETS-1 in nuclear and the recruitment of ETS-1 to its targeted genes’ promoter, it is likely that activated ERα may interact with ETS-1 and induce its translocation into nuclear and recruit each other onto their DNA binding sites. Further time-effect or dose-effect experiments should be done to further discover the mechanism of estrogen/ERα on ETS-1 cytoplasm/nuclear translocation.

The ETS family includes a large number of transcriptional regulatory proteins. All ETS family members share an 85 amino acid conserved DNA binding domains (ETS domain) in the C-terminal of the protein [35]. They may play compensatory roles in physiological, pharmaceutical and pathological regulation of growth, migration, invasion, apoptosis and oncogenic transformation [36] process. Thus, we cannot exclude the possibility that ERα also interacts with other ETS family members, such as ETS-2. It is valuable to examine the cross-talk of ERα with other members of ETS1 family besides ETS-1.

Although ERα was detected in endocrine-related cancers, besides to breast cancer, the function of ERα need to be further discovered. ERα inhibitor or antagonist, ICI-182780 or tamoxifen would inhibit the growth of breast cancer, HCC, neuroblastoma, and glioma cells [37]. It’s well known that ERα associates with some other signaling pathways [5, 6, 38]. Jiang et al., 2013 showed that protein MEMO mediated the interaction of HER2 and ERα [38]. Egloff et al., 2009 reported that estrogen increased transcription from ERE and induced activation of MAPK in HNSCC cell lines [4]. In spite of those accumulating discoveries, whether ERα plays a role in neuroblastoma oncogenesis is still unknown. Our work extended the understanding of ERα function and it is necessarily to further learn the roles of cross-talk of ERα with relative signaling pathways in neuroblastoma cells.

The proliferation, invasion and migration are the main features of the metastatic malignancies, which are markers in cancer progression and are major causes of mortality. Recent data showed that several important genes participated in the regulation of cancer cells’ proliferation. To date, a subset of patients would suffer from the tumor with ERα positively expressing, such as HCC, neuroblastoma and ovarian cancer. In this work, based on the previous data, we choose SH-SY5Y as a neuroblastoma cell model. Estrogen treatment enhanced the proliferation, anchor-independent growth, invasion and migration of ERα-positive neuroblastoma cell SH-SY5Y and up-regulated the transcriptional activity of ETS-1. Thus, we deduced that estrogen level would be a novel bio-marker or risk factor in the prognosis of neuroblastoma, and the anti-endocrine therapies targeted to ERα would be a novel strategy of neuroblastoma treatment.

## Conclusions

In summary, estrogen/ERα is involved in neuroblastoma proliferation and enhanced the activation of ETS-1. This notion is supported by the fact that E2 treatment enhanced the transcription factor activity of ETS-1 through promoting ERα/ETS-1 interaction. Here, we demonstrate that the interaction of ERα and ETS-1 participates in regulation of neuroblastoma cell’s proliferation, migration and invasion in the presence of estrogen. These findings would help us to understand more about E2/ERα signaling in cancerous cell proliferation and also provide a new potential therapeutic target of human neuroblastoma.

## Abbreviations

ERα: Estrogen receptor α
HCC: Hepatocellular Carcinoma
HNSCC: Head and neck squamous cell carcinoma
ETS-1: E26 transformation specific sequence 1
co-IP: Co-immunoprecipitation
ChIP: Chromatin-immunoprecipitation
ERE: Estrogen responsive element
EBS: ETS-binding sites
HGF: Hepatocyte growth factor
SRC-1: Steroid receptor coactivator 1
AIB-1: Amplified in breast cancer1
AF-1 domain: Activation function domain 1
AF-2: Activation function domain 2
DBD domain: DNA binding domain
MAPK: Mitogen-activated protein kinase
MMP1/9: Matrix metalloproteinase1/9
EGFR: Epidermal growth factor receptor
ECM: Extracellular matrix
AHR: Aryl hydrocarbon receptor
